# Pediatric triage variations among nurses, pediatric and emergency residents using the Canadian triage and acuity scale

**DOI:** 10.1186/s12873-021-00541-0

**Published:** 2021-11-22

**Authors:** Saleh Alshaibi, Tala AlBassri, Suliman AlQeuflie, Winnie Philip, Nesrin Alharthy

**Affiliations:** 1 Collage of Medicine, King Saud bin Abdulaziz University for Health Science, Riyadh, Saudi Arabia; 2 Department of Pediatrics, King Abdulaziz Medical City, Riyadh, Saudi Arabia; 3 Research Unit, College of Applied Medical Sciences, King Saud bin Abdulaziz University for Health Science, Riyadh, Saudi Arabia; 4 Department of Pediatrics Emergency, Emergency Department- King Abdulaziz Medical City, King Abdullah International Medical Research Center, Riyadh, Saudi Arabia

**Keywords:** CTAS, Triaging system, Pediatrics

## Abstract

**Background:**

Emergency care continues to be a challenge, since patients’ arrival is unscheduled and could occur at the same time which may fill the Emergency Department with non-urgent patients. Triaging is an integral part of every busy ED. The Canadian Triage and Acuity Scale (CTAS) is considered an accurate tool to be used outside Canada. This study aims to identify the chosen triage level and compare the variation between registered nurses, pediatric and adult emergency residents by using CTAS cases.

**Method:**

This study was conducted at King Abdulaziz Medical City,Saudi Arabia. A cross-sectional self-administered questionnaire was used, and which contains 15 case scenarios with different triage levels. All cases were adopted from a Canadian triage course after receiving permission. Each case provides the patient’s symptoms, clinical signs and mode of arrival to the ED. The participants were instructed to assign a triage level using the following scale. A non-random sampling technique was used for this study. The rates of agreement between residents were calculated using kappa statistics (weighted-kappa) (95%CI).

**Result:**

A total of 151 participants completed the study questionnaire which include 15 case scenarios. 73 were nurses and 78 were residents. The results showed 51.3, 56.6, and 59.9% mis-triaged the cases among the nurses, emergency residents, and pediatric residents respectively. Triage scores were compared using the Kruskal Wallis test and were statistically significant with a *p* value of 0.006. The mean ranks for nurses, emergency residents and pediatric residents were 86.41, 73.6 and 59.96, respectively. The Kruskal Wallis Post-Hoc test was performed to see which groups were statistically significant, and it was found that there was a significant difference between nurses and pediatrics residents (*P* value = 0.005). Moreover, there were no significant differences found between nurses and ER residents (P value> 0.05).

**Conclusion:**

The triaging system was found to be a very important tool to prioritize patients based on their complaints. The results showed that nurses had the greatest experience in implementing patients on the right triage level. On the other hand, ER and pediatric residents need to develop more knowledge about CTAS and become exposed more to the triaging system during their training.

## Background

The emergency department (ED) is one of the most crucial sectors of the healthcare system. Emergency care continues to be a challenge, since patients’ arrival is unscheduled with different disease acuity. There has been a steady increase in the number of patients visiting EDs annually, which leads to a rise in resource utilization. Studies have shown that about 25–60% of the patients seeking care in emergency departments are real emergency cases [1, 2]. However, EDs may become filled with non-urgent patients, leading to overcrowding. Overcrowded EDs affect the patient in terms of resources and adequate care in a timely fashion due to overwhelming ER staff physicians [3]. Furthermore, prioritizing and making a decisive decision about the severity of a condition will allow allocation of resources to sick patients in timely manner [4].

Triage is a French word originating in the late 1700s, which means “to sort” or “to select “. In the past, it was used to sort injured soldiers by prioritizing them according to the type and urgency of their conditions [5, 6]. Today, triaging is an integral part of every busy emergency department. It ensures that patients receive appropriate attention in response to their clinical needs.

Over several years, many formalized systems appeared, which are used differently worldwide. These include the Australian Triage Scale (ATS), the Canadian Triage and Acuity Scale (CTAS), the Manchester Triage System (MTS), and the Emergency Severity Index (ESI) [7]. In Saudi Arabia, there is still no unified triage system. However, CTAS has been applied for more than 10 years in different hospitals in Saudi Arabia, including King Abdullah Specialist Children’s Hospital (KASCH) [8]. Although the Canadian Triage and Acuity Scale (CTAS) was first utilized in Canada, several studies showed that CTAS is considered an accurate and reliable tool for patient assessment outside of Canada.

CTAS is a triaging system used in ED to facilitate patient evaluation process, communication between nurses and physicians as well as resources allocation. Several studies concluded the outstanding validity and reliability of the CTAS [9–12]. In 2001, a modified guidelines were developed and published for evaluation of pediatric emergencies [13, 14]. According to CTAS, triage is classified into five levels based on patients’ initial evaluations. Critically ill patients that need a resuscitation immediately will be triage as level 1. On the other hand, patients who are not expected to deteriorate in short period of time will be triage based on presenting clinical complaints (triage level 2–5). In 2004, the Canadian Emergency Department Information System (CEDIS) developed a standardized list of complaints based on 2 modifiers [15]. First order modifiers, which include level of consciousness,pain severity and mechanism of injury [13]. As well as vital signs acceptable for age, which is an important modifier in triaging patients [16]. One SD above or below the age relevant normal is classified CTAS 3; 2 SD, CTAS 2; 3 SD, CTAS 1. Patients vital signs in the normal range are CTAS 4 or 5 [17]. Second order modifiers, are specific to number of complains which is used after first order modifiers did not assign patients to any triage level [15].

During a short assessment, nurses identify signs and symptoms that determine the patient’s urgency. The role of triaging nurses is vital for assessing the patient, such as communicating with the public, communicating with health professionals, assigning resources, initiating treatment protocols/first aid measures, monitoring and reassessing, participating in patient flow, and documenting [18]. A study conducted among nurses with at least 6 months of triage experience showed that there is moderate to low agreement among nurses in determining the level of triage for pediatric patients [5]. After a short assessment, physicians see patients in order of their urgency level and manage them accordingly. Also, there are some differences between physicians in triaging experience based on their specialties. This has been shown in several areas, including the management of febrile seizure and sedation use [19]. However, many studies have focused only on the management of cases, and not on physicians’ or nurses’ perceptions of triaging patients [20].

Several studies have been conducted in pediatrics triaging systems, specifically CTAS. However, limited studies focused on pediatrics populations or CTAS in Saudi Arabia [21]. The objective of this study is to determine whether differences exist between pediatric, adult nurses and residents in triaging pediatric patients.

## Methods

A cross-sectional design using a questionnaires survey to assess participants knowledge about patient triage using clinical scenarios in a period between 2018 and 2020. This study was conducted at King Abdulaziz Medical City, Saudi Arabia. A non-probability convenience sample was used, as we were conducting the study data collection during a clinical shift, and the responders were therefore selected based on their accessibility and time. Based on an estimate, we have around 80 residents in both emergency and pediatric residency programs. We targeted all, since we expected a response rate of around 80%. Nurses were selected based on the known population size of 200 and 5% as a margin of error with a 95% confidence interval, and 50% as the response distribution, and the calculated sample size of 132. A rough estimate of 200 participants was considered for the study. All residents were included have a mandatory pediatric ED rotation which is a core rotation during their residency program based on SCFHS. Pediatrics and adult emergency nurses were also included since they are mandated to take course of CTAS course. Residents in the study institution are supervised by senior ER physician during the rotation in ER in all area including Triage area. We excluded emergency fellows and emergency consultants from the study due to expected high knowledge, which would have affected the study results.

A questionnaire of 15 case scenarios was distributed among nurses and residents. All cases were adopted from the CTAS course. Permission was obtained from the a CATS course developer in order to use the course case scenarios. The cases were reviewed and readjusted culturally and environmentally. Moreover, cases that could be triaged into two different levels were eliminated to avoid confusion. We included two to three from each level to reflect reality.

The case scenarios described patients coming to the ED. Each case provided background information on the patients, symptoms, important clinical signs, and mode of arrival to the ED. The participants were instructed to assign a triage level using the following scale: 1 = resuscitation, 2 = emergent, 3 = urgent, 4 = less urgent, and 5 = non-urgent (Table 1). All the data collected were entered using Microsoft Excel. The data were analyzed using SPSS version 22 software. Differences between the three groups. A *p*-value of less than 0.05 was considered statistically significant. Results were expressed in tables and figures. Mean and Standard Deviation (SD) were used for continuous variables. The rates of agreement between pediatrics, adult residents and nurses were calculated using kappa statistics (weighted kappa) (95% CI). The following guidelines for the interpretation of kappa statistics were used: less than 0.40, poor to fair; 0.41 to 0.60, moderate; 0.61 to 0.80, substantial; and greater than 0.80, almost perfect.

**Table 1:** CTAS time objectives

Triage level Time for triage	Time to nursing assignment	Time to nursing reassessment	Time to physician assessment
I	≤10 min	Continuous care	Immediate
II		Every 15 min	≤15 min
III		Every 30 min	≤30 min
IV		Every 60 min	≤60 min
V		Every 120 min	≤120 min

## Results

Of the 151 participants (73 Nurses, 36 ER residents and 42 pediatric residents) a 100% response rate was achieved for all case scenarios. The mean (± SD) age was 31.14 (± 6.76) years with the majority, 66%, being females. Among the 73 nurses, 29 (40%) were working in pediatrics and 44 (60%) were working with adults. For the ED residents, 36.2% were in level R2, 22.2% were in levels R1 and R4 and 19.4% were in level R3. Similarly for pediatric residents, a greater part of them, 50%, were in level R4, while 23.8% were in R2, 21.4% were in R3 and 4.8% were in R1.

**Fig. 1:**
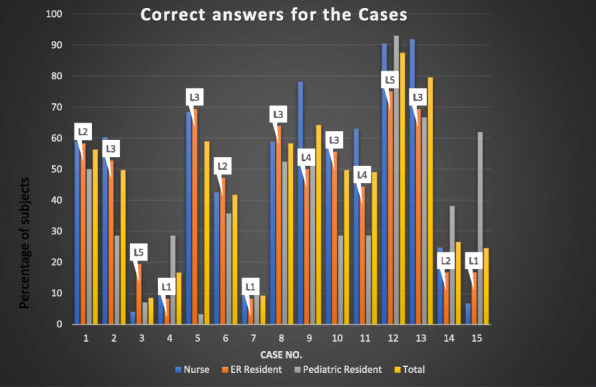
Percentage of correct answers for the 15 Case scenarios between the groups with the correct triage level mentioned in the Fig. (L = level)

Among pediatric residents 38.1% had used the CTAS to triage pediatric patients. Fifty-five percent of the pediatric residents knew about CTAS scales. Of the pediatric residents, all had performed an emergency rotation (42, 100%). In addition, comparing cases answers between the 3 groups, showing that the nurses have the most corrected answers as demonstrated in the Fig. 1. However, pediatric residents have had higher levels of achievements of correct answers in triaging patients with level 1 and, they are less confident triaging patients in level 3. This finding was not statically significate.

**Fig. 2:**
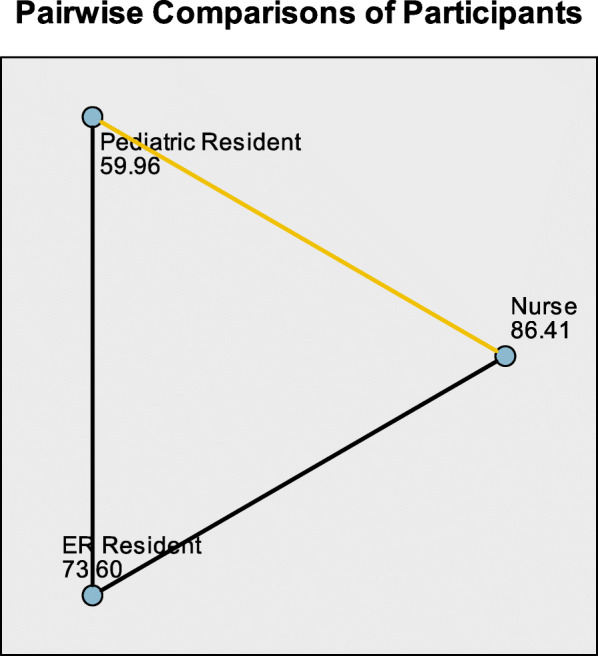
Post Hoc Tests for the triage score and the participants

Triage scores were compared using the Kruskal Wallis test and were statistically significant with a *p* value of 0.006. The mean ranks for nurses, emergency residents and pediatric residents were 86.41, 73.6 and 59.96, respectively (Table 2). Figure 2 describes the Kruskal Wallis Post Hoc test and shows that the scores were statistically significant among pediatric residents (mean rank = 59.96) and nurses (mean rank = 86.41) with a *p* value of 0.005. The scores were statistically insignificant for pediatric residents-emergency residents (*p* value = 0.493) and for emergency resident-nurses (*p* value = 0.435) (Fig. 2).

**Table 2:** Comparing the Triage score among Nurses, Pediatric residents and emergency Residents

Participants	No. of subjects	Mean Rank	Test used & Test Statistic	*P* value
Nurse	73	86.41	Kruskal Wallis Test Chi square = 10.158	0.006*
Emergency Resident	36	73.60		
Pediatric Resident	42	59.96		
Total	151			

*Statistically significant at 5%

Weighted Kappa along with a 95% confidence interval was used to find the level of agreement among nurses and residents based on the level of training. The overall agreement levels for residents were moderate, with the weighted Kappa as 0.438. The level of agreement was observed to be good among the nurses, with weighted Kappa as 0.688. Among the pediatric residents (Weighted Kappa = − 1.455) and emergency residents (Weighted Kappa = − 0.470), the agreement level was observed to be poor (Table 3).

**Table 3:** Level of agreement among Nurses and Residents (Pediatric and Emergency) with Weighted Kappa and 95% CI

	No. of subjects	Weighted Kappa	95% CI
Residents	78	0.438	(−0.282–0.832)
Pediatric Residents	42	−1.455	(−2.375–0.932)
Emergency Residents	36	−0.470	(−1.556–0.577)
Nurses	73	0.688	(−0.077–1.00)
Nurses and Residents	151	0.193	(−0.213–0.473)

## Discussion

Triage is a very important tool in emergency departments, which requires the whole team to be aware of in order to standardize the care of health care providers while handling patients and promote proper utilization of resources. Non-urgent cases are estimated to be 9–54% in the United States of America, and 25.5–60% in Canada [1, 22]. A well-structured triaging system and multidisciplinary team recommended to be implemented in each emergency department for optimum patient care and crowd control. The level of agreement among nurses was satisfactory, which indicates the strength of training and experience in the field of patient triaging. In contrast, patient triaging was not an integral part of the resident training program, which was reflected in the study findings.

The results showed 51.3, 56.6, and 59.9% mis-triaged the cases based on the standard scenarios adopted from CTAS for nurses, Emergency residents, and pediatric residents, respectively, which may negatively impact patient care. ED overcrowding affects the hospital financially [20]. Over-triaging can have a huge impact on hospital resources allocation and timely patient care. A study conducted among pediatrics injured patients found that 61% of the patients who were considered to have a severe trauma were discharged from the pediatrics center in less than 24 h [23]. On the other hand, under-triage can harm the patient by delaying management and putting the patient at risk of deterioration, especially for the pediatric age group, which might be accompanied by unclear signs of serious illness [22, 23].

Inter-rater agreement using the CTAS scale has been studied for many years, mostly among nurses. Dallaire et al. compared nurses based on their experience and found a moderate agreement among them (weighted kappa of 0.44) [24]. This finding contradicts our result, in which we compared a number of senior and junior nurses with different years of experience and in which practice found a good agreement between nurses, with a weighted kappa of 0.688. However, our study’s results were similar to those of Alquraini et al., which also found good agreement (weighted kappa of 0.770) [8]. In addition, upon comparison between pediatrics residents, poor agreement was found (weighted kappa of − 1.455) in this study. In contrast, a moderate agreement was concluded by Bergeron et al. (weighted kappa of 0.419) [5]. In terms of physicians compared to nurses, this study resulted in a poor level of agreement (weighted kappa 0.193). Unlike our finding, a meta-analysis of 12 studies showed a pooled estimator of a good level of agreement with a value of 0.797 [25]. Unsurprisingly, nurses performed better than residents in our study, due to their daily experience and regular practice of using a triaging scale.

Study main limitations that we used case based scenarios instead of real ED patients. Even though the cases in this study were adopted from the CTAS courses with some cultural modifications, few studies support that triage reliability testing can be tested using case scenarios as an alternative to real patients [26]. The data presented in this study comes from only one institution. This could affect the ability to generalize our findings to other facilities. Since our center is considered one of the largest emergency trauma centers in the region, we believe that our setting is ideal for testing CTAS reliability. We also used a limited number of scenarios, and all of them were pediatrics populations. We specifically chose the more common scenarios seen in our region. Since adult Emergency residents mandatorily rotate under pediatric ED, we included only pediatric cases.

## Conclusion

Patients can present with a broad spectrum of emergencies that range from minor to severe presentations. In order to avoid overcrowding, having a well-structured triaging system is crucial to every ED. Also, nurses and physicians need to be more aware of how to use and apply this system. Our results showed that nurses had the highest level of experience in implementing patients on the right triage level, due to their knowledge and practice with CTAS scale. Additionally, these results signify the importance of emergency and pediatric residents to become familiar and have enough exposure to and training with the triaging system..

## Data Availability

The datasets used and/or analyzed during the current study are available from the corresponding author on reasonable request.

## Abbreviations

ED: Emergency Department
ATS: Australian Triage Scale
CTAS: Canadian Triage and Acuity Scale
MTS: Manchester Triage System
ESI: Emergency Severity Index
KASCH: King Abdullah Specialist Children’s Hospital
CEDIS: Canadian Emergency Department Information System
SD: Standard Deviation
